# Striatal Transcriptome and Interactome Analysis of *Shank3*-overexpressing Mice Reveals the Connectivity between Shank3 and mTORC1 Signaling

**DOI:** 10.3389/fnmol.2017.00201

**Published:** 2017-06-28

**Authors:** Yeunkum Lee, Sun Gyun Kim, Bokyoung Lee, Yinhua Zhang, Yoonhee Kim, Shinhyun Kim, Eunjoon Kim, Hyojin Kang, Kihoon Han

**Affiliations:** ^1^Department of Neuroscience, College of Medicine, Korea UniversitySeoul, South Korea; ^2^Department of Biomedical Sciences, College of Medicine, Korea UniversitySeoul, South Korea; ^3^Center for Synaptic Brain Dysfunctions, Institute for Basic Science (IBS)Daejeon, South Korea; ^4^Department of Biological Sciences, Korea Advanced Institute of Science and Technology (KAIST)Daejeon, South Korea; ^5^HPC-enabled Convergence Technology Research Division, Korea Institute of Science and Technology InformationDaejeon, South Korea

**Keywords:** *Shank3*, mTORC1, striatum, mania, Rhes

## Abstract

Mania causes symptoms of hyperactivity, impulsivity, elevated mood, reduced anxiety and decreased need for sleep, which suggests that the dysfunction of the striatum, a critical component of the brain motor and reward system, can be causally associated with mania. However, detailed molecular pathophysiology underlying the striatal dysfunction in mania remains largely unknown. In this study, we aimed to identify the molecular pathways showing alterations in the striatum of SH3 and multiple ankyrin repeat domains 3 (Shank3)-overexpressing transgenic (TG) mice that display manic-like behaviors. The results of transcriptome analysis suggested that mammalian target of rapamycin complex 1 (mTORC1) signaling may be the primary molecular signature altered in the *Shank3* TG striatum. Indeed, we found that striatal mTORC1 activity, as measured by mTOR S2448 phosphorylation, was significantly decreased in the *Shank3* TG mice compared to wild-type (WT) mice. To elucidate the potential underlying mechanism, we re-analyzed previously reported protein interactomes, and detected a high connectivity between Shank3 and several upstream regulators of mTORC1, such as tuberous sclerosis 1 (TSC1), TSC2 and Ras homolog enriched in striatum (Rhes), via 94 common interactors that we denominated “Shank3-mTORC1 interactome”. We noticed that, among the 94 common interactors, 11 proteins were related to actin filaments, the level of which was increased in the dorsal striatum of *Shank3* TG mice. Furthermore, we could co-immunoprecipitate Shank3, Rhes and Wiskott-Aldrich syndrome protein family verprolin-homologous protein 1 (WAVE1) proteins from the striatal lysate of *Shank3* TG mice. By comparing with the gene sets of psychiatric disorders, we also observed that the 94 proteins of Shank3-mTORC1 interactome were significantly associated with bipolar disorder (BD). Altogether, our results suggest a protein interaction-mediated connectivity between Shank3 and certain upstream regulators of mTORC1 that might contribute to the abnormal striatal mTORC1 activity and to the manic-like behaviors of *Shank3* TG mice.

## Introduction

Bipolar disorder (BD), characterized by recurrent mood swings between depression and mania, is a highly heritable and chronic mental illness that affects approximately 2.5% of the population worldwide (Merikangas et al., 2011). Manic episodes are the defining feature of BD, and manic symptoms include hyperactivity, impulsivity, elevated mood, reduced anxiety and decreased need for sleep (Grande et al., 2016). These core symptoms suggest that a dysfunction of the striatum, the key component of the brain motor and reward systems, may be involved in the pathogenesis of manic disorder. This hypothesis is supported by structural and functional abnormalities observed in the striatum of the patients with BD (Strakowski et al., 1999; Blumberg et al., 2003; Wessa et al., 2007). However, the detailed molecular pathophysiology underlying striatal dysfunction in mania remains largely unknown.

The mammalian target of rapamycin (mTOR) pathway integrates various external signals and controls diverse cellular processes including translation, apoptosis, autophagy and energy metabolism (Laplante and Sabatini, 2012). The serine/threonine kinase mTOR forms two protein complexes, mTOR complex 1 (mTORC1) and mTORC2, which have different subunit compositions and cellular functions. The heterodimeric complex of tuberous sclerosis 1 (TSC1) and TSC2 is a critical upstream regulator of mTORC1 that functions as a guanosine triphosphatase (GTPase)-activating protein (GAP) for the small GTPase Ras homolog enriched in brain (Rheb; Huang and Manning, 2008). As the active (GTP-bound) form of Rheb directly binds and activates mTORC1, TSC1/TSC2 complex is a negative regulator of mTORC1 pathway. In the striatum, Ras homolog enriched in striatum (Rhes, encoded by *Rasd2* gene), a small GTPase highly enriched in the striatal medium spiny neurons (MSNs), has roles similar to Rheb in directly binding and activating mTORC1 in a GTP-dependent manner (Subramaniam et al., 2011). The activity of Rhes is regulated by Ras guanyl releasing protein 1 (RasGRP1), a guanine nucleotide exchange factor (GEF), in the striatum (Shahani et al., 2016).

In the brain, the mTOR pathway is involved in various aspects of neuronal development and function including dendrite formation, axonal elongation and synapse formation and plasticity (Hoeffer and Klann, 2010; Takei and Nawa, 2014). This pathway has critical roles in normal brain function, as abnormalities in the expression and/or activity of its upstream and downstream components have been identified in numerous neurodevelopmental and neuropsychiatric disorders, including autism spectrum disorders (ASDs), drug addiction, intellectual disability (ID), major depressive disorder (MDD), and schizophrenia (SCZ; Costa-Mattioli and Monteggia, 2013). Specifically, it has been shown that mTORC1 pathway is compromised in the prefrontal cortex of patients with MDD (Jernigan et al., 2011). Furthermore, the therapeutic efficacy of a fast-acting antidepressant ketamine is dependent on the activation of mTORC1 pathway that increases the synthesis of excitatory synaptic proteins (such as PSD-95 and glutamate receptors) and the number of dendritic spines in the prefrontal cortex (Li et al., 2010; Abdallah et al., 2015). However, potential alterations of the mTOR pathway in the striatum of the patients with mania have been scarcely investigated.

Several pharmacological and genetic rodent models of mania have been generated and characterized, and these, even with some limitations, have provided important insights towards understanding the pathogenic mechanisms in mania (Chen G. et al., 2010; Kato et al., 2016; Logan and McClung, 2016). We recently reported that *EGFP-Shank3* (SH3 and multiple ankyrin repeat domains 3)-overexpressing transgenic (TG) mice display manic-like behaviors at the adult stage (8 to 12-week-old), such as locomotor hyperactivity, hypersensitivity to amphetamine, increased acoustic startle response, reduced prepulse inhibition and abnormal circadian rhythms. Although some of the behavioral abnormalities of *Shank3* TG mice could also be observed in mice modeling other disorders such as ASDs and SCZ, the *Shank3* TG mice responded to valproic acid, a Food and Drug Administration (FDA)-approved drug for the treatment of manic or mixed episodes in BD (Han et al., 2013b). The *Shank3* TG mice mildly overexpress Shank3 proteins (by approximately 50%) compared to wild-type (WT) mice, and thus, could potentially model human patients with *SHANK3* gene duplications who usually have an additional copy of *SHANK3* gene. Indeed, we could also identify several patients with *SHANK3* gene duplications who were diagnosed with mania-like hyperkinetic disorders (Han et al., 2013b). These results altogether support the construct, face and predictive validity (Nestler and Hyman, 2010) of *Shank3* TG mice to model human mania. However, importantly, it needs to be validated whether the *SHANK3* duplication patients with mania-like hyperkinetic disorders indeed express higher Shank3 protein levels. It is also notable that *SHANK3* duplications have been identified in patients with some other disorders including Asperger’s syndrome, SCZ, and attention deficit hyperactivity disorder (ADHD; Durand et al., 2007; Failla et al., 2007; Moessner et al., 2007). In addition to the behavioral phenotypes, we also showed abnormalities of synaptic actin cytoskeleton and dendritic spines in the hippocampus of *Shank3* TG mice (Han et al., 2013b). Nevertheless, the hippocampus might not be the primary brain region mediating manic-like behaviors of *Shank3* TG mice, especially considering that Shank3 is enriched in the striatum compared to other brain regions (Peça et al., 2011; Monteiro and Feng, 2017). Moreover, the identity of downstream signaling pathways that may be affected by altered synaptic actin cytoskeleton in *Shank3* TG mice remains uninvestigated.

*SHANK3* (also called *ProSAP2* for proline-rich synapse-associated protein 2) gene encodes a core scaffold protein organizing the macromolecular protein complex of the neuronal excitatory postsynapse (Sheng and Kim, 2000; Dosemeci et al., 2016). In addition to duplications, deletions and various point mutations of *SHANK3* gene have been causally associated with ASDs, ID and SCZ (Durand et al., 2007; Gauthier et al., 2010; Grabrucker et al., 2011; Jiang and Ehlers, 2013; Guilmatre et al., 2014; Leblond et al., 2014; Choi et al., 2015; Monteiro and Feng, 2017). Notably, several studies investigating *Shank3* knock-out (KO) mice and *Shank3* knock-down in neurons revealed that the loss of Shank3 expression results in changes of specific signaling pathways such as metabotropic glutamate receptor 5 (mGluR5) signaling (Verpelli et al., 2011; Wang et al., 2016; Vicidomini et al., 2017), and protein kinase B (PKB/Akt)-mTOR signaling (Bidinosti et al., 2016). Moreover, pharmacological treatments (mGluR5 modulators, insulin-like growth factor 1 (IGF1), or Cdc2-like kinase 2 (CLK2) inhibitor) targeting these pathways recued both synaptic and behavioral abnormalities caused by the loss of Shank3 expression (Bozdagi et al., 2013; Shcheglovitov et al., 2013; Bidinosti et al., 2016; Wang et al., 2016; Vicidomini et al., 2017). Therefore, it is conceivable that such signaling pathways might also be altered especially in the striatum of *Shank3* TG mice, which could potentially provide an insight into the molecular pathophysiology underlying the striatal dysfunction in manic-like behaviors.

To address this issue, in this study, we performed a transcriptome (RNA sequencing) analysis on the striatal tissue of adult WT and *Shank3* TG mice, the results of which suggested mTORC1 signaling as the primary molecular signature affected by *Shank3* overexpression. Based on the transcriptome analysis, we examined mTORC1 activity in the striatum of *Shank3* TG mice and found that it was decreased compared to that in WT mice. To understand the potential underlying mechanisms, we re-analyzed the previously reported protein interactome data, which revealed that 94 interactors were shared between Shank3 and upstream regulators of mTORC1 (TSC1, TSC2 and Rhes). Moreover, we found that several of the 94 common interactors were involved in regulating actin filaments (F-actin), the amount of which was increased in the dorsal striatum of *Shank3* TG mice. By performing a comparison with the disease-associated gene sets, we found that these 94 common interactors were significantly associated with BD and SCZ, but not ASDs. Altogether, our results suggest a protein interaction-mediated connectivity between Shank3 and certain upstream regulators of mTORC1 that might contribute to the abnormal striatal mTORC1 activity and, at least in part, to the manic-like behaviors of *Shank3* TG mice.

## Materials and Methods

### Mice

The enhanced green fluorescent protein (EGFP)-*Shank3* TG mice used in this study have been described previously (Han et al., 2013b; Lee B. et al., 2017; Lee Y. et al., 2017). The WT and *Shank3* TG mice were bred and maintained on a C57BL/6J background according to the Korea University College of Medicine Research Requirements, and all procedures were approved by the Committees on Animal Research at Korea University College of Medicine (KOREA-2016-0096). The mice were fed *ad libitum* and housed under a 12-h light-dark cycle.

### RNA Sequencing and Analysis

The mice (12-week-old male WT and *Shank3* TG, three mice per genotype) were deeply anesthetized with isoflurane and decapitated. The striatum was dissected from each brain using a brain matrix, immediately placed in RNAlater solution (Ambion), and stored at 4°C overnight. RNA extraction, library preparation, cluster generation, and sequencing were performed by Macrogen Inc. (Seoul, Korea). RNA samples for sequencing were prepared using a TruSeq RNA Sample Prep Kit v2 (Illumina) according to the manufacturer’s instructions. An Illumina’s HiSeq 2000 was used for sequencing to generate 101-bp paired-end reads (Supplementary Table S1). Raw data were submitted to the Gene Expression Omnibus (GEO) repository with accession GSE97544.

Pre-processing of raw reads was carried out using Trimmomatic (Bolger et al., 2014) (version 0.35, options: LEADING:3 TRAILING:3 MAXINFO:80:0.4 MINLEN:36), and the trimmed reads were mapped to the *Mus musculus* genome (GRCm38) using TopHat2 (Kim D. et al., 2013; version 2.1.0, default options). The gene-level read counts were calculated from the aligned reads using HTSeq Python package (Anders et al., 2015). Differential gene expression analysis was performed using DEseq2 package in R/Bioconductor (Love et al., 2014). Normalized read counts were computed by dividing the raw read counts by size factors and fitted to a negative binomial distribution. The *P* values were first corrected by applying an empirical estimation of the null distribution using the R fdrtool (v.1.2.15) package and then adjusted for multiple testing with the Benjamini–Hochberg correction. Genes with an adjusted *P* value of less than 0.05 were considered as differentially expressed.

Gene Set Enrichment Analysis (GSEA^1^; Subramanian et al., 2005) was used to determine whether a *priori*-defined gene sets would show statistically significant differences in expression between *Shank3* TG and WT mice. Enrichment analysis was performed using GSEAPreranked (gsea2-2.2.2.jar) module on gene set collections H (Hallmark gene sets; 50 gene sets) downloaded from Molecular Signature Database (MSigDB) v5.1^2^. Additionally, GSEA analysis was performed by using the sets of genes associated with psychiatric disease from the Psychiatric disorders Gene association NETwork (PsyGeNET) database (last update: Sept., 2016; Gutierrez-Sacristan et al., 2015). GSEAPreranked was applied using the list of all genes expressed, ranked by the fold change and multiplied by the inverse of the *P* value with recommended default settings (1000 permutations and a classic scoring scheme). The False Discovery Rate (FDR) was estimated to control the false positive finding of a given Normalized Enrichment Score (NES) by comparing the tails of the observed and null distributions derived from 1000 gene set permutations. The gene sets with an FDR of less than 0.05 were considered as significantly enriched.

### Construction of Interactome Network

To build an interaction network, the sets of Shank3 (Han et al., 2013b), TSC1/TSC2 (Sakai et al., 2011) and Rhes (Shahani et al., 2016) interactomes were adopted. The network graphics were generated using Cytoscape (Shannon et al., 2003). To simplify the network, orphan nodes, defined as the nodes connecting with only one of the hub proteins (Shank3, TSC1/TSC2 and Rhes), were excluded from the graphics.

### Gene Ontology (GO) and Kyoto Encyclopedia of Genes and Genomes (KEGG) Pathway Analysis

The Gene Ontology (GO) and Kyoto Encyclopedia of Genes and Genomes (KEGG) pathway analyses were performed using DAVID software (version 6.8; Huang Da et al., 2009). The set of 94 genes from Shank3-mTORC1 interactome was tested against a customized background of the entire mouse genome. Mouse gene names were converted to human homologs using the Mouse Genome Informatics (MGI) database^3^.

### Disease Association Analysis

Gene-disease association data were retrieved from the PsyGeNET (Gutierrez-Sacristan et al., 2015) and Disease gene association NETwork (DisGeNET) databases (Piñero et al., 2015; last update: Sept., 2016). PsyGeNET database contains information relevant to psychiatric diseases and their associated genes integrated from the DisGeNET (Piñero et al., 2017) database, and data extracted from the literature by text mining, and further curated by the experts in the domain. Additionally, the genes associated with the risk for ASD were obtained from the Simons Foundation Autism Research Initiative (SFARI) database (syndromic and category 3 or above^4^). The enrichment of disease-associated genes was tested using the hypergeometric distribution test. Hypergeometric *P* values were calculated using the phyper (q: overlapped genes-1, m: Shank3-mTORC1 interactome, n: protein-coding genes in The HUGO Gene Nomenclature Committee (HGNC)—m, k: disease associated genes) function in R package, and were adjusted for multiple testing with the Benjamini and Hochberg test, as implemented in the Bioconductor’s *q* value package. Diseases with adjusted *P* values of less than 0.05 were considered as statistically significantly enriched.

### RNA Purification and qRT-PCR

Real-time quantitative reverse transcription PCR (qRT-PCR) was performed as described previously (Han et al., 2013a; Kim et al., 2016; Lee B. et al., 2017). Briefly, total RNA was extracted from the striatum of 12-week-old mice using a miRNeasy minikit (Qiagen) according to the manufacturer’s instructions. Two micrograms of total RNA were used for cDNA synthesis using iScript^TM^ cDNA Synthesis Kit (Bio-Rad). Target mRNAs were detected and quantified by a real-time PCR instrument (CFX96 Touch, Bio-Rad) using SYBR Green master mix (Bio-Rad). The results were analyzed using the comparative Ct method normalized against the housekeeping gene *Gapdh*. The primer sequences for real-time PCR are as follows:

Mouse *Shank3* forward 5′ TGGTTGGCAAGAGATCCAT 3′,
   reverse 5′ TTGGCCCCATAGAACAAAAG 3′
Mouse *Gpr85* forward 5′ ATGCAGCCGACAACATTTTGC 3′,
   reverse 5′ CAGGTGGAGCCATTTTTGACA 3′
Mouse *Clic6* forward 5′ CTCTGGGTTAGACTCTCAGGG 3′,
   reverse 5′ GGTGCCTCTGTGTCCATGTT 3′
Mouse *Plk5* forward 5′ CGGCACCCTTGTCAGAGATG 3′,
   reverse 5′ TGGGGGAAAGGCAAACACAG 3′
Mouse *Gapdh* forward 5′ GGCATTGCTCTCAATGACAA 3′,
   reverse 5′ CCCTGTTGCTGTAGCCGTAT 3′

### Biochemistry and Antibodies for Western Blotting

Whole lysates of the mouse brain were prepared as described previously (Han et al., 2009, 2015). Briefly, the striatum and hippocampus of 12-week-old mice were homogenized in RIPA buffer (50 mM Tris-HCl pH 8.0, 150 mM NaCl, 0.1% SDS, 1% Triton X-100, 0.5% sodium deoxycholate) with freshly added protease and phosphatase inhibitors (Roche). Protein concentration was measured using Bradford Protein Assay (Bio-Rad). Brain lysates were heated in 1x NuPAGE LDS sample buffer (Invitrogen) containing a 1x NuPAGE reducing agent (Invitrogen). From each sample, 10~20 μg of proteins were loaded for Western blotting. Immunoprecipitation (IP) was performed as described previously (Han et al., 2013b; Lee Y. et al., 2017). The GFP-Trap beads (ChromoTek) were used to pull down EGFP-Shank3 proteins and their interactors. The antibodies used for Western blotting were Gapdh (Cell Signaling, #2118), GFP (NeuroMAb, #75-131), Homer1b/c (Santa Cruz, sc-20807), Rhes (Millipore, ABN31), Shank3 (Santa Cruz Biotechnology, sc-30193), phospho-mTOR (S2448, Cell Signaling, #2971), mTOR (Cell Signaling, #2983), and WAVE1 (NeuroMab, 75-048). Western blot images were acquired by ChemiDoc Touch Imaging System (Bio-Rad) and quantified using ImageJ software.

### Immunohistochemistry and Image Analysis

For each immunohistochemistry (IHC) experiment, 5–7 pairs of 12-week-old WT and *Shank3* TG mice from at least three different litters were used. The mice were deeply anesthetized with isoflurane and transcardially perfused with heparinized (20 units/ml) phosphate-buffered saline (PBS) followed by 4% paraformaldehyde (PFA) in PBS. Brains were extracted and post-fixed in 4% PFA overnight. After post-fixation, the brains were washed with PBS and cryoprotected with 30% sucrose in PBS for 48 h. Brains were frozen in O.C.T compound (SAKURA Tissue-Tek, 4583) and sectioned (60 μm) using a cryostat microtome (Leica, CM3050S). For each staining set, two sections were randomly selected from each mouse at a similar anterior posterior level. The following antibodies were used: GFP (Abcam, ab290, 1:500), neuronal nuclei (NeuN; Millipore, MAB377, 1:1000), rhodamine phalloidin (Molecular Probes, R415, 1 unit/200 μl), and Alexa Fluor-conjugated secondary antibodies (Jackson Immunoresearch, 111-585-003 and 115-585-003, 1:500). Confocal microscopy (Zeiss, LSM800) was used to acquire images (10× objective and 0.6× digital zoom) of the striatum (Bregma 0.13–1.7) from coronal sections. Whole regions were obtained by tile scanning and each frame was taken in Z-stacks of 5–10 slices (in total 45–55 μm thickness). Tiled Z-projection images were aligned and converted into a single flattened image using ZEN software from Zeiss. From each tiled image, randomly selected two regions of interests (ROIs) were analyzed for the dorsolateral (DL), dorsomedial (DM), and dorsoventral (DV; ventral part of the dorsal striatum) striatum using ImageJ software. All quantifications were carried out by operators blinded to the genotype.

### Quantification and Statistical Analysis

Values from at least three independent experiments using were used for quantification and statistical analysis. This means that we performed at least three independent technical experiments, and we used different biological samples for each technical experiment. *P* values were calculated by two-tailed unpaired Student’s *t*-test unless otherwise specified, using GraphPad Prism 6 software. All data are presented as mean ± SEM. *P < 0.05; **P < 0.01; ***P < 0.001.

## Results

### Striatal Transcriptome Analysis of *Shank3* TG Mice

**Figure 1:**
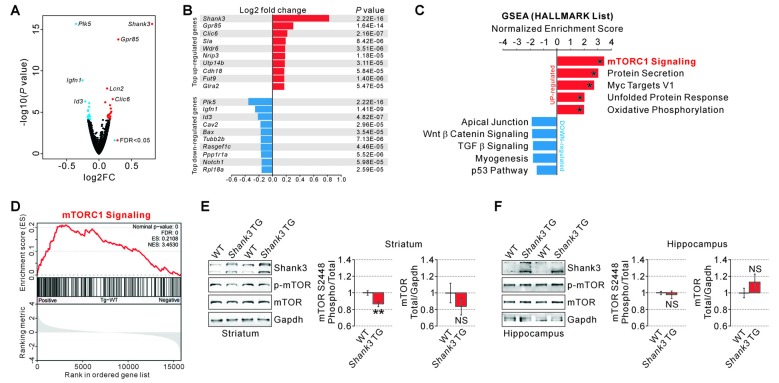
RNA-seq analyses and measurements of mammalian target of rapamycin complex 1 (mTORC1) activity in the SH3 and multiple ankyrin repeat domains 3 (*Shank3*) transgenic (TG) striatum. **(A)** Volcano plot for the striatal RNA-seq analysis of *Shank3* TG mice. Differentially expressed genes (DEGs), defined by false discovery rate (FDR) < 0.05, are shown as blue (down-regulated) and red (up-regulated) circles. FC, fold change. The complete lists of RNA-seq analysis and DEGs are provided in the Supplementary Tables S2, S3. **(B)** List of top 10 up-regulated and down-regulated DEGs (based on the fold changes) from the striatal RNA-seq analysis of *Shank3* TG mice. **(C)** The bar graph shows normalized enrichment scores (NES) of gene set enrichment analysis (GSEA) on the Hallmark gene sets for the striatal RNA-seq analysis of *Shank3* TG mice. Asterisks indicate the gene sets with an FDR of less than 0.05. The complete list that contains the results of GSEA analysis is provided in Supplementary Table S4. **(D)** The enrichment plot of striatal RNA-seq analysis of *Shank3* TG mice on the mTORC1 signaling gene set. The complete list of mTORC1 signaling genes within the *Shank3* TG RNA-seq analysis is provided in Supplementary Table S5. **(E)** Representative Western blot images and quantifications show that mTORC1 activity measured by mTOR S2448 phosphorylation is decreased in the striatum of *Shank3* TG mice. The total mTOR expression level is not significantly different between wild-type (WT) and *Shank3* TG striatum. Data are presented as mean ± SEM (*n* = 10 animals per genotype; **P < 0.01, unpaired two-tailed Student’s *t*-test). **(F)** Normal mTORC1 activity and total mTOR protein levels in the hippocampus of *Shank3* TG mice (*n* = 6 animals per genotype).

To identify which signaling pathways were altered in the striatum of *Shank3* TG mice, we performed transcriptome (RNA sequencing, RNA-seq) analysis with the striatal tissue from 12-week-old WT and *Shank3* TG mice. Although most Shank3 proteins function at the excitatory postsynapse, and thus, are less likely to directly regulate broad gene transcription, we reasoned that this unbiased approach might (indirectly) reflect major signaling pathways affected by mild Shank3 overexpression. As expected, the overall changes in gene expression were mild in the striatum of *Shank3* TG mice compared to that of WT mice (Figure 1A and Supplementary Table S2). After applying adjusted *P* values (<0.05, Benjamini–Hochberg correction), we could identify 22 up-regulated and 17 down-regulated genes in the *Shank3* TG striatum (Supplementary Table S3); *Shank3* (1.77 fold), G protein-coupled receptor 85 (*Gpr85*, 1.23 fold), and Chloride intracellular channel 6 (*Clic6*, 1.16 fold) were the top three up-regulated genes, while Polo like kinase 5 (*Plk5*, −1.28 fold), Immunoglobulin-like and fibronectin type III domain containing 1 (*Igfn1*, −1.19 fold), and Inhibitor of DNA binding 3 (*Id3*, −1.16 fold) were the top three down-regulated genes (Figure 1B), some of which were validated by qRT-PCR (Supplementary Table S3 and Supplementary Figure S1).

Next, we performed GSEA to identify biologically meaningful signatures in the *Shank3* TG mice striatal RNA-seq data. We found that several biological pathways including “mTORC1 signaling”, “protein secretion”, “Myc targets V1”, “unfolded protein response (UPR)”, and “oxidative phosphorylation” were represented by the genes up-regulated in *Shank3* TG striatum (Figure 1C and Supplementary Table S4). Among them, “mTORC1 signaling” was the top ranked pathway based on the NES (Figures 1C,D and Supplementary Tables S4, S5). Moreover, protein secretion, UPR, and oxidative phosphorylation are the cellular processes tightly coupled with mTORC1 signaling (Narita et al., 2011; Appenzeller-Herzog and Hall, 2012; Morita et al., 2013), which further supports the hypothesis that mTORC1 signaling may be the primary molecular signature in the RNA-seq analysis of *Shank3* TG striatum. In contrast to the up-regulated genes, the genes down-regulated in *Shank3* TG striatum depicted no significant enrichment in any specific biological pathways (Figure 1C and Supplementary Table S4).

Based on the results from GSEA, we directly investigated the striatal mTORC1 activity in *Shank3* TG mice by measuring the phosphorylation level of mTOR S2448 residue. The phosphorylation of mTOR S2448 is predominantly associated with mTORC1 (Copp et al., 2009), and reflects its activation as S6 kinase, a downstream target of mTORC1, phosphorylates the residue in a feedback loop (Chiang and Abraham, 2005). We found that the phosphorylation level of mTOR S2448 normalized to total mTOR expression was significantly decreased by approximately 15% in the striatum of *Shank3* TG mice compared to that of WT mice (Figure 1E). The total expression level of mTOR protein was not significantly altered in the striatum of *Shank3* TG mice (Figure 1E). In contrast to the striatum, neither phospho nor total mTOR protein was altered in the hippocampus of *Shank3* TG mice (Figure 1F). Altogether, these results suggest that mild overexpression of Shank3 decreases mTORC1 activity in the striatum of *Shank3* TG mice.

### Protein Interactome Analysis of Shank3 with the Upstream Regulators of mTORC1

What could be the mechanism underlying decreased mTORC1 activity in the striatum of *Shank3* TG mice? The serine/threonine kinase PKB/Akt is a key positive upstream regulator of mTORC1 that directly phosphorylates and inhibits the TSC1/TSC2 complex (Laplante and Sabatini, 2012). However, in our original report describing the *Shank3* TG mice, we showed that the activities of PKB/Akt and one of its downstream targets, glycogen synthase kinase 3 (GSK3), were normal in the striatum of *Shank3* TG mice (Han et al., 2013b). Therefore, we decided to explore alternative targets.

**Figure 2:**
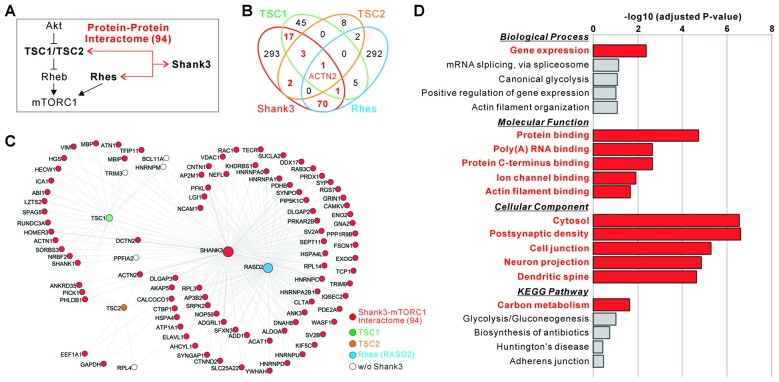
Protein interaction-mediated connectivity between Shank3 and upstream regulators of mTORC1. **(A)** The diagram shows a summary of protein interaction-mediated connectivity between Shank3 and the upstream regulators of mTORC1 (tuberous sclerosis 1, TSC1/TSC2 and Ras homolog enriched in striatum, Rhes). **(B)** The Venn diagram shows the number of interactors shared by Shank3, TSC1, TSC2 and Rhes proteins. The actin filament bundling protein, α-actinin 2 (ACTN2), is the only common interactor for all four proteins. **(C)** The interactome network of Shank3, TSC1/TSC2 and Rhes. To simplify the network, the orphan nodes, defined as nodes connected with only one of the hub proteins (Shank3, TSC1/TSC2 and Rhes), are excluded from the graphics. The nodes for 94 proteins of Shank3-mTORC1 interactome are colored in red. The complete list of interactomes for Shank3, TSC1, TSC2 and Rhes is provided in Supplementary Table S6. **(D)** Gene ontology (GO) and kyoto encyclopedia of genes and genomes (KEGG) pathway analyses of the 94 proteins of Shank3-mTORC1 interactome. Significant terms (Benjamini adjusted *P* value < 0.05) are in bold red. The complete list of analyses is provided in Supplementary Table S7.

We have previously generated a comprehensive Shank3 protein interactome consisting of about 400 proteins, by combining the results from yeast two-hybrid (Y2H) screening (Sakai et al., 2011) and *in vivo* IP followed by mass spectrometry analysis of the mixed hippocampal and striatal tissue isolated from *Shank3* TG mice (Han et al., 2013b). As Shank3 is a core scaffold protein containing multiple protein-protein interaction (PPI) domains, it is possible that the functions of Shank3 in neurons could be largely mediated by the interacting proteins (Lee Y. et al., 2017). Notably, Sakai et al. (2011) have previously provided evidence supporting the PPI-mediated connectivity between Shank3 and mTORC1 pathway. Specifically, they showed that Shank3 and TSC1/TSC2 complex are highly connected by many interacting proteins identified by Y2H screening, and that Shank3 and TSC1 indeed form an *in vivo* protein complex in the mouse brain. In addition, Shahani et al. (2016) recently published a striatal *in vivo* protein interactome of Rhes, another upstream regulator of mTORC1, (“Rhesactome”) where Shank3 was identified as a Rhes interactor in the striatum. Therefore, we re-analyzed these interactomes (Shank3 *in vivo* + Y2H, TSC1 and TSC2 Y2H, and Rhes *in vivo*) side by side to elucidate the identity and the number of proteins interacting both with Shank3 and the upstream regulators of mTORC1. Notably, we found an overlap consisting of 94 proteins (about 24%) between the Shank3 interactome and either TSC1, TSC2, or Rhes interactome (Figures 2A–C and Supplementary Table S6). Among the 94 proteins, four interactors including Dynactin 2 (encoded by *Dctn2*), Ankyrin repeat domain 35 (Ankrd35), Pleckstrin homology like domain family B member 1 (Phldb1), and Protein interacting with C kinase 1 (Pick1) were shared by three proteins (either Shank3/TSC1/TSC2 or Shank3/TSC1/Rhes), and one interactor, α-actinin 2 (encoded by *Actn2*), was shared by all four proteins (Figures 2B,C).

To understand the representative biological functions or pathways of the 94 common interactors, we performed GO and KEGG pathway analysis. We found that terms including “gene expression” in the biological process category, “protein binding”, “poly(A) RNA binding”, “ion channel binding”, and “actin filament binding” in the molecular function category, and “cytosol”, “postsynaptic density”, “neuronal projection”, and “dendritic spine” in the cellular component category, and “carbon metabolism” in the KEGG pathway were significantly associated with these interactors (Figure 2D and Supplementary Table S7). Together, these results suggest a high connectivity between Shank3 and the upstream regulators of mTORC1, mediated by 94 common interacting proteins involved in defined biological pathways (for simplicity, we will refer to these 94 proteins as “Shank3-mTORC1 interactome”).

### Actin-Related Proteins in the Shank3-mTORC1 Interactome

**Figure 3:**
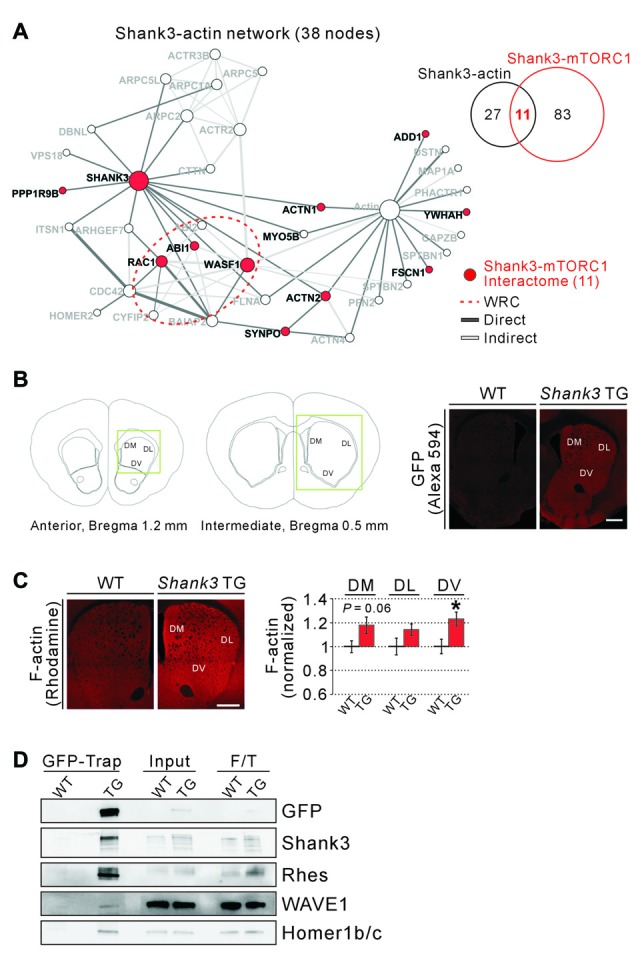
Actin-related proteins in the Shank3-mTORC1 interactome, and increased F-actin levels in the *Shank3* TG striatum. **(A)** The actin-related subnetwork of Shank3 interactome where the 11 proteins shared by the Shank3-mTORC1 interactome are colored in red. The edges of the network are colored depending on the type of interactions (direct or indirect interaction). The red dotted circle indicates the components of wave regulatory complex (WRC; WASF1; Abelson-interacting protein 1 (ABI1), ABI2 and Cytoplasmic FMR1-interacting protein 2 (CYFIP2) and Ras-related C3 botulinum toxin substrate 1 (RAC1)). **(B)** The diagrams show the atlas of mouse brain coronal sections for the striatum, and the coordinates of the exact areas where immunohistochemistry (IHC) experiments were performed (left panel). The green boxes indicate regions of interests (ROIs). Representative IHC images show expression of the EGFP-Shank3 proteins in the dorsal striatum of *Shank3* TG, but not WT, mice (right panel). DL, dorsolateral; DM, dorsomedial; DV, dorsoventral. Scale bar, 500 μm. **(C)** Representative IHC images and quantification show increased levels of F-actin in the DV, but not DM and DL, compartments of *Shank3* TG striatum. Scale bar, 500 μm. Data are presented as mean ± SEM (*n* = 7 animals per genotype; *P < 0.05, unpaired two-tailed Student’s *t*-test). **(D)** Western blot images show co-immunoprecipitation (IP) of EGFP-Shank3, Rhes, WAVE1, and Homer1b/c proteins from the *Shank3* TG, but not WT, synaptosomal lysate. For input and flow-through (F/T) lanes, 0.5% of total proteins were loaded.

From the GO and KEGG pathway analysis, we could identify several molecular/biological functions of the 94 proteins from Shank3-mTORC1 interactome. Among them, we hypothesized that actin regulatory pathway might be important, because ACTN2, an actin filament bundling protein (Djinovic-Carugo et al., 1999), was identified as the only common interactor for Shank3, TSC1/TSC2, and Rhes. Moreover, PICK1, a common interactor for Shank3 and TSC1/TSC2, is also involved in regulating actin cytoskeleton by inhibiting the actin-nucleating Arp2/3 complex (Rocca et al., 2008). Previously, we generated a subnetwork of Shank3 interactome consisting of only 38 actin-related proteins from the original Shank3 interactome (Han et al., 2013b). We found that among the 38 actin-related interactors of Shank3, 11 proteins (approximately 29%) were shared with the Shank3-mTORC1 interactome (Figure 3A). Specifically, we noticed that the Wiskott-Aldrich syndrome protein family verprolin-homologous protein 1 (WAVE1, encoded by *WASF1*) and Abelson-interacting protein 1 (Abi1) proteins, the critical components of the WAVE regulatory complex (WRC), and Ras-related C3 botulinum toxin substrate 1 (Rac1), the upstream regulator of the WRC, were among these 11 proteins (Figure 3A). The WRC is an approximately 400 kDa heteropentameric protein complex that consists of WAVE, Abi, Cytoplasmic FMR1-interacting protein (Cyfip), Nck-associated protein (Nap) and Hematopoietic stem progenitor cell 300 (HSPC300) and activates the Arp2/3 complex to initiate actin polymerization and branching (Takenawa and Suetsugu, 2007; Chen Z. et al., 2010; Han et al., 2015).

This result prompted us to test whether the levels of polymerized actin (actin filament or F-actin) were increased in the striatum of *Shank3* TG mice. We previously showed that synaptic F-actin levels were increased in the cultured hippocampal neurons of *Shank3* TG mice (Han et al., 2013b). However, this has not been validated *in vivo*, especially in the striatum where Shank3 is enriched compared to other brain regions. For the analysis, we focused on the dorsal striatum, the area in which the functional and morphological changes of striatal synapses in the *Shank3* KO mice have been mainly characterized (Peça et al., 2011; Peixoto et al., 2016; Wang et al., 2016; Jaramillo et al., 2017). In addition, the dorsal striatum is closely associated with motor and executive functions (Balleine and O’Doherty, 2010) both of which are defective in mania (Marvel and Paradiso, 2004). Nevertheless, to more precisely characterize F-actin changes, we subdivided the dorsal striatum into three subareas; DL, DM and DV compartments, characterized by distinct cellular compositions, synaptic inputs/outputs, and functional roles in controlling behavior (Steiner and Tseng, 2010; Ito and Doya, 2015; Matamales et al., 2016; Figure 3B).

First, we carefully set the scanning parameters for fluorescent confocal microscopy, because *Shank3* TG mice express EGFP-tagged Shank3 proteins. Indeed, we found that under the scanning condition for Alexa Fluor 488, significant amount of signal was detected from the striatum of *Shank3* TG, but not WT, mice (Supplementary Figure S2). Therefore, we decided to use Alexa Fluor 594 and rhodamine for IHC experiments. Next, we confirmed that the EGFP-Shank3 proteins from the transgene were expressed in all three striatal subareas of *Shank3* TG mice (Figure 3B). Lastly, we measured F-actin levels by staining the striatal sections with rhodamine-conjugated phalloidin, and found that F-actin levels were significantly increased by approximately 23% in the DV compartment of *Shank3* TG mice (Figure 3C). Trends for an increase were also observed in DL (*P* = 0.14) and DM (*P* = 0.06) compartments, but the differences were not statistically significant. As a control, the intensity of NeuN staining in the three subareas of the dorsal striatum in *Shank3* TG mice was comparable to that in WT mice, suggesting a normal neuronal density in the *Shank3* TG striatum (Supplementary Figure S3).

Previously, Sakai et al. (2011) validated the *in vivo* interactions of Shank3, TSC1 and α-actinin using mouse brain lysates. Therefore, we also tested for the existence of the *in vivo* protein complex consisting of Shank3, Rhes and WAVE1 in the synaptosomal lysate from *Shank3* TG mice. We performed IP using the GFP-Trap beads to pull down EGFP-Shank3 and its interacting proteins from *Shank3* TG mice, as described previously (Han et al., 2013b; Lee Y. et al., 2017), and found that Shank3, Rhes, WAVE1 and Homer1b/c (a known interactor of Shank3) proteins were pulled down together (Figure 3D). This result is consistent with the previous study of “Rhesactome”, in which the authors performed IP using Rhes antibodies to pull down endogenous Rhes proteins from the striatal lysate of WT mice, and identified Shank3 and WAVE1 proteins in the complex using mass spectrometry analysis (Shahani et al., 2016). Together, these results suggest that certain actin-related proteins may be involved in connecting Shank3 and the upstream regulators of mTORC1.

### Associations of the Shank3-mTORC1 Interactome and *Shank3* TG Transcriptome with the Disease

Recent studies have shown that the genes mutated in neurodevelopmental and neuropsychiatric disorders such as ASD or SCZ might also be highly interconnected at the protein level (De Rubeis et al., 2014; Fromer et al., 2014). In this regard, we reasoned that neuropsychiatric disorders affected by pathological Shank3-mTORC1 interactions might be identified as those significantly associated with the 94 proteins connecting Shank3 and the upstream regulators of mTORC1. We selected three established disease-associated gene sets; psychiatric disorder-associated PsyGeNET gene sets (Gutierrez-Sacristan et al., 2015), ASD-associated Simons Foundation Autism Research Initiative (SFARI) gene set^5^, and broad human disease-associated DisGeNET gene sets (Piñero et al., 2017) and compared these gene sets with the 94 genes of Shank3-mTORC1 interactome.

**Figure 4:**
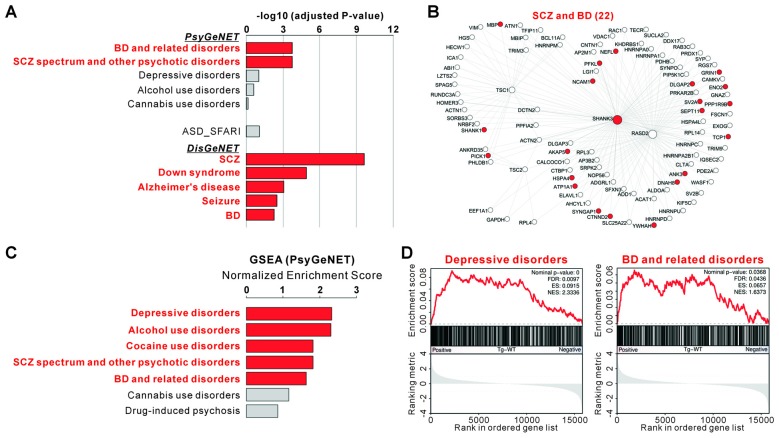
Associations of the Shank3-mTORC1 interactome and *Shank3* TG striatal transcriptome with the disease. **(A)** Disease association analysis for the 94 genes in Shank3-mTORC1 interactome. Significant disease terms (Benjamini adjusted *P* value < 0.05) are in bold red. The complete analyses are provided in Supplementary Tables S8, S9. **(B)** The Shank3-mTORC1 interactome network with bipolar disorder (BD)- and schizophrenia (SCZ)-associated proteins from Psychiatric disorders Gene association NETwork (PsyGeNET) database colored in red. **(C)** The bar graph shows the Normalized Enrichment Scores (NESs) of GSEA on the PsyGeNET gene sets for the striatal RNA-seq analysis of *Shank3* TG mice. Significant disease terms are in bold red. The complete list of GSEA is provided in Supplementary Table S10. **(D)** The enrichment plots showing the striatal RNA-seq analysis of *Shank3* TG mice on the PsyGeNET gene sets of “depressive disorders” (left panel) and “BD and related disorders” (right panel).

We found that among the eight major classes of psychiatric disorders in the PsyGeNET, “BDs and related disorders” and “SCZ spectrum and other psychiatric disorders” were significantly associated with the 94 genes of Shank3-mTORC1 interactome (adjusted *P* = 0.000186 for both, hypergeometric test; Figures 4A,B and Supplementary Table S8). Moreover, when compared with the entire subclasses (117 psychiatric disorders) of PsyGeNET, only four disorders including “SCZ”, “BD”, “manic” and “manic mood” were significantly associated with the 94 genes (Supplementary Table S8). It is considered that BD and SCZ have many aspects in common, such as risk genes, molecular and neuronal pathophysiology, and clinical symptoms (Berrettini, 2000; Benes and Berretta, 2001; Moskvina et al., 2009). In contrast to BD and SCZ, the association between ASDs (SFARI genes) and the 94 genes was not statistically significant (Figure 4A). Lastly, among the disease classes of DisGeNET, “SCZ”, “Down syndrome”, “Alzheimer’s disease”, “seizures” and “BD” were among the top disorders significantly associated with the 94 genes of Shank3-mTORC1 interactome (Figure 4A and Supplementary Table S9).

The above mentioned result showing a significant association between Shank3-mTORC1 interactome and BD prompted us to test whether the overall transcriptomic change in the *Shank3* TG striatum might also be associated with BD or other psychiatric disorders. We therefore preformed GSEA of the *Shank3* TG RNA-seq results with the PsyGeNET gene sets. We found that more PsyGeNET gene sets were significantly associated with the RNA-seq results than with the Shank3-mTORC1 interactome. In addition to SCZ and BD, “depressive disorders”, “alcohol use disorders”, and “cocaine use disorders” gene sets were significantly represented by the genes up-regulated in the *Shank3* TG striatum (Figures 4C,D and Supplementary Table S10). Together, these results suggest that both Shank3-mTORC1 interactome and striatal transcriptome of *Shank3* TG mice may be associated with BD.

## Discussion

The aim of this study was to elucidate the molecular pathophysiology underlying striatal dysfunction in mania. We performed striatal RNA-seq analysis of *Shank3*-overexpressing manic mouse model and identified mTORC1 signaling as the primary molecular signature. Based on the RNA-seq analysis, we examined mTORC1 activity and found that it was decreased in the striatum of *Shank3* TG mice. Although the RNA-seq analysis revealed altered mTORC1 activity, we consider that the gene expression changes related to mTORC1 signaling could be a secondary or compensatory effect of decreased mTORC1 activity for several reasons. First, the GSEA revealed that mTORC1 signaling was depicted by the genes up-regulated in *Shank3* TG striatum, which is opposite to the change of striatal mTORC1 activity. Second, Shank3 proteins mainly function at the excitatory postsynapse, and are thus less likely to directly regulate a group of genes specifically related to mTORC1 signaling. Notably, it was recently reported that Shank3 proteins can undergo synapse-to-nucleus shuttling in an activity-dependent manner, and that Shank3 localized in the nucleus may regulate the expression of several genes such as leucine rich repeat transmembrane neuronal 1 (*Lrrtm1*), synaptotagmin I (*Syt1*), and cystic fibrosis transmembrane conductance regulator homolog (*Cftr*; Grabrucker et al., 2014). However, none of these potential “Shank3 target genes” was listed among mTORC1 signaling-related genes from our RNA-seq analysis (Supplementary Table S5), suggesting that mTORC1 signaling-related genes in the striatum of *Shank3* TG mice were not directly up-regulated by nuclear Shank3 proteins.

Although we did not characterize them in details, there were several interesting differentially expressed genes (DEGs) from the striatal RNA-seq analysis of *Shank3* TG mice. For example, an up-regulated *GPR85* (also called *SREB2* for super-conserved receptor expressed in brain) gene, encoding a highly conserved G protein-coupled receptor, has been associated with SCZ (Matsumoto et al., 2008; Radulescu et al., 2013). Notably, the *Gpr85* TG mice mildly overexpressing GPR85 proteins display some abnormal behaviors including impaired prepulse inhibition and decreased social interaction (Matsumoto et al., 2008), which were also seen in the *Shank3* TG mice (Han et al., 2013b). Therefore, the effects from DEGs, together with decreased mTORC1 activity, might contribute to the behavioral phenotypes of *Shank3* TG mice.

To understand the detailed mechanism underlying decreased mTORC1 activity in the *Shank3* TG striatum, we re-analyzed the Shank3, TSC1/TSC2 and Rhes protein interactomes side by side. This was based on the previous reports showing the PPI-mediated connectivity between Shank3 and TSC1/TSC2 (Sakai et al., 2011), and the striatal *in vivo* Rhes protein complex (“Rhesactome”) containing Shank3 and several Shank3-interacting proteins (Shahani et al., 2016). Indeed, we could identify 94 proteins connecting Shank3 and the upstream regulators of mTORC1 (Shank3-mTORC1 interactome). However, one of the limitations of this interactome is not being strict enough to fully represent the *in vivo* connections among Shank3, TSC1/TSC2 and Rhes, because some of the interactions, especially those for TSC1/TSC2, were identified based only on *in vitro* Y2H screening. Nevertheless, Shank3-TSC1 (Sakai et al., 2011) and Shank3-Rhes (Figure 3D) interactions were validated in the mouse brain lysates, indicating the existence of protein complexes containing Shank3, TSC1/TSC2 and Rhes *in vivo*.

The mechanism underlying the decrease in mTORC1 activity in response to interactions between Shank3 and the upstream regulators of mTORC1 in the *Shank3* TG striatum remains unclear. One possibility is that Shank3 overexpression might shift or sequester TSC1/TSC2 and Rhes proteins from mTORC1 regulatory complex to actin filaments-related complex and thereby disturb the maintenance of normal mTORC1 activity. In support of this hypothesis, we found that 11 of the 94 proteins from Shank3-mTORC1 interactome, including ACTN2, WAVE1, Abi and Rac1, were associated with actin filaments, and that levels of F-actin were increased in the dorsal striatum of *Shank3* TG mice. Similarly, it was recently shown that, in the mouse striatum, RasGRP1 suppresses the inhibitory role of Rhes in amphetamine-induced dopamine receptor signaling, by promoting Rhes to form a specific protein complex (Shahani et al., 2016). Further biochemical and/or imaging analyses are necessary to fully understand the functional significance of Shank3-mTORC1 interactome in the regulation of the striatal mTORC1 activity. Moreover, we cannot exclude the possibility that altered mTORC1 activity might contribute to the increased F-actin levels in the striatum of *Shank3* TG mice. It has been shown that mTORC1 and mTORC2 regulate motility and metastasis of colorectal cancer cells via modulating Ras homolog gene family, member A (RhoA) and Rac1 signaling (Gulhati et al., 2011).

It is notable that mTORC1 activity is also decreased in the neurons with reduced Shank3 expression, due to increased steady-state levels of CLK2 (Bidinosti et al., 2016). The CLK2 phosphorylates and activates the regulatory subunit of protein phosphatase 2A (PP2A) which in turn inactivates Akt, a positive regulator of mTORC1. However, this mechanism could not explain the decrease in mTORC1 activity in the striatum of *Shank3* TG mice, where Akt activity was found to be normal (Han et al., 2013b). Thus, either loss or gain of Shank3 expression can induce a decrease in mTORC1 activity, most likely, via different mechanisms, the details of which will be an interesting topic for future research. Despite normal Akt activity in the *Shank3* TG striatum, however, treatments with certain molecules that increase Akt activity such as IGF1, might possibly rescue the decreased striatal mTORC1 activity and some behavioral abnormalities in the *Shank3* TG mice. Importantly, the treatment with IGF1 has already been shown as a promising potential therapeutic approach for the disorders caused by *SHANK3* deficiency in model system studies and a pilot clinical trial (Bozdagi et al., 2013; Shcheglovitov et al., 2013; Kolevzon et al., 2014; Bidinosti et al., 2016).

By performing Western blot experiments, we found a mild (approximately 15%) decrease of mTORC1 activity in the striatum of *Shank3* TG mice. While several types of neurons can be found in the striatum, MSNs, the GABAergic output neurons of the striatum, account for the majority (>90%) of the population. The MSNs can be further classified into D1 and D2-type neurons based on the type of dopamine receptor expressed and projection pathway (striatonigral direct pathway and striatopallidal indirect pathway, respectively; Calabresi et al., 2014). It has been reported that the excitatory synapses of MSNs in *Shank3* KO mice show morphological and functional abnormalities (Peça et al., 2011; Peixoto et al., 2016; Wang et al., 2016; Jaramillo et al., 2017). However, these studies did not address whether D1 and D2-type MSNs could be differentially affected by the loss of Shank3 expression. Importantly, Wang et al. (2017) recently showed that several striatal synaptic functions are selectively impaired in the striatopallidal D2-type MSNs in a line of *Shank3* KO (*Shank3B* KO) mice. In the same regard, the striatal mTORC1 activity of *Shank3* TG mice may be preferentially, or even specifically, decreased in the D1 or D2-type MSNs, but this decrease may be masked by normal mTORC1 activity of the remaining neuronal populations. More comprehensive analyses regarding the activities of mTORC1 in different neuronal populations of the striatum of both *Shank3* KO and TG mice will be important, especially considering that D1 and D2-type MSNs of the striatum have distinct, or even opposite, roles in controlling behavior (Kravitz et al., 2010).

Another important remaining issue is whether decreased striatal mTORC1 activity is causally associated with manic-like behaviors in *Shank3* TG mice. This issue could be addressed by testing the effects of drugs, such as IGF1, that can increase mTORC1 activity on the manic-like behaviors of *Shank3* TG mice. In this case, however, treatment during the development may be critical given the possibility that the decrease in mTORC1 activity starts during early development and chronically affects the striatum to finally result in an adult onset of manic-like behaviors. This is possible because at least Shank3 expression itself is increased starting at juvenile (approximately 3-week-old) stage in the *Shank3* TG mice compared to WT mice (Han et al., 2013b).

We found that, unlike in the striatum, mTORC1 activity was normal in the hippocampus of *Shank3* TG mice. However, we cannot exclude the possibility that mTORC1 activities in other brain regions, such as frontal cortex and cerebellum, could be altered and contribute to manic-like behaviors of *Shank3* TG mice. Interestingly, it has been shown that intracerebroventricular injection of ouabain, an inhibitor of Na/K-ATPase, increases Akt and mTORC1 activities in the frontal cortex and causes manic-like behaviors in rats (Yu et al., 2010; Kim S. H. et al., 2013). Therefore, mTORC1 activities of different brain regions might have distinct, or even opposite, roles in causing manic-like behaviors. Even with these remaining issues, our bioinformatic analysis showed that both the genes belonging to Shank3-mTORC1 interactome and the up-regulated transcriptome of *Shank3* TG striatum were significantly associated with the PsyGeNET BD genes (Figure 4). Therefore, investigating the role of these Shank3-related BD genes, together with mTORC1 signaling, in *Shank3* TG mice might enable us to fully understand the molecular mechanisms of manic-like behaviors in these mice.

In conclusion, our results suggest a decrease in mTORC1 activity in the striatum of *Shank3* TG mice that may be potentially mediated by the PPI-dependent connectivity between Shank3 and several upstream regulators of mTORC1. Decreased striatal mTORC1 activity might contribute to manic-like behaviors in *Shank3* TG mice, but further investigation is needed to validate such hypothesis. Since both Shank3 and mTORC1 signaling are implicated in a broad spectrum of neurodevelopmental and neuropsychiatric disorders, the connectivity between these two proteins may be an important converging pathophysiological pathway as well as a potential therapeutic target for the treatment of various brain disorders.

## Author Contributions

YL, SGK, BL, YZ, YK, SK and KH designed and performed the experiments. HK and KH analyzed and interpreted the data. EK discussed the project and provided reagents. YL, HK and KH wrote the article. All authors read and approved the manuscript.

## Conflict of Interest Statement

The authors declare that the research was conducted in the absence of any commercial or financial relationships that could be construed as a potential conflict of interest.

## Footnotes

^1^http://software.broadinstitute.org/gsea

^2^http://software.broadinstitute.org/gsea/msigdb

^3^http://www.informatics.jax.org/homology.shtml

^4^https://gene.sfari.org/autdb/GS_Home.do

^5^https://gene.sfari.org/autdb/GS_Home.do

## Supplementary Material

FIGURE S1 Validation of differentially expressed genes in the striatum of *Shank3* TG mice. The graph shows the results of qRT-PCR experiments validating an increase in the expression of *Shank3*, *Gpr85* and *Clic6*, and a decrease in the expression of *Plk5* in the striatum of *Shank3* TG mice compared to WT mice. Data are presented as mean ± SEM (*n* = 4–6 animals per genotype; **P* < 0.05, ****P* < 0.001, unpaired two-tailed Student’s *t*-test).

FIGURE S2 Detection of EGFP signal from the striatum of *Shank3* TG mice. To test whether there was any background signal from the striatal sections of WT and *Shank3* TG mice, we performed IHC experiments only with the secondary antibodies (Alexa Fluor 488 and 555). Under the scanning condition for Alexa Fluor 488, significant amount of signal (potentially from the EGFP-Shank3 proteins) was detected from the striatum of *Shank3* TG, but not WT, mice. Meanwhile, no signal was detected for Alexa Fluor 555 from both WT and TG striatum. DIC, differential interference contrast.

FIGURE S3 Normal NeuN intensity in the dorsal striatum of *Shank3* TG mice. Representative IHC images and quantification show normal NeuN intensity in the DM, DL and DV compartments of *Shank3* TG striatum. Scale bar, 500 μm. DL, dorsolateral; DM, dorsomedial; DV, dorsoventral. Data are presented as mean ± SEM (*n* = 5 animals per genotype; *P* > 0.05, unpaired two-tailed Student’s *t*-test).
