# Supplementary figures and images for: Striatal Transcriptome and Interactome Analysis of Shank3-overexpressing Mice Reveals the Connectivity between Shank3 and mTORC1 Signaling
Source: Front Mol Neurosci. 2017 Jun 28;10:201. doi: 10.3389/fnmol.2017.00201 (PMC5487420; doi:10.3389/fnmol.2017.00201)

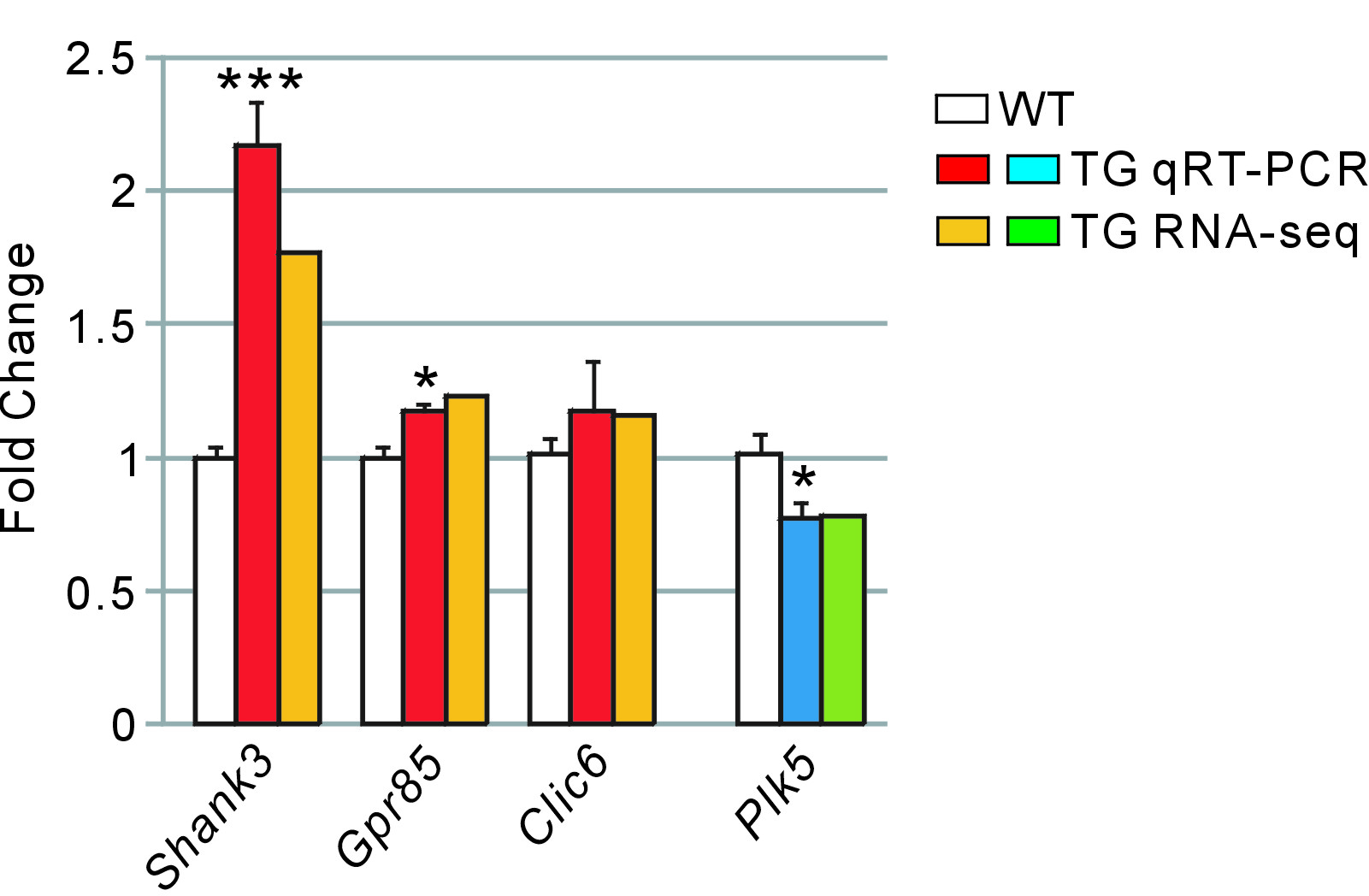

Supplement: FIGURE S1 — Validation of differentially expressed genes in the striatum of Shank3 TG mice. The graph shows the results of qRT-PCR experiments validating an increase in the expression of Shank3, Gpr85 and Clic6, and a decrease in the expression of Plk5 in the striatum of Shank3 TG mice compared to WT mice. Data are presented as mean ± SEM (n = 4–6 animals per genotype; *P < 0.05, ***P < 0.001, unpaired two-tailed Student’s t-test). [file Image_1.jpeg]

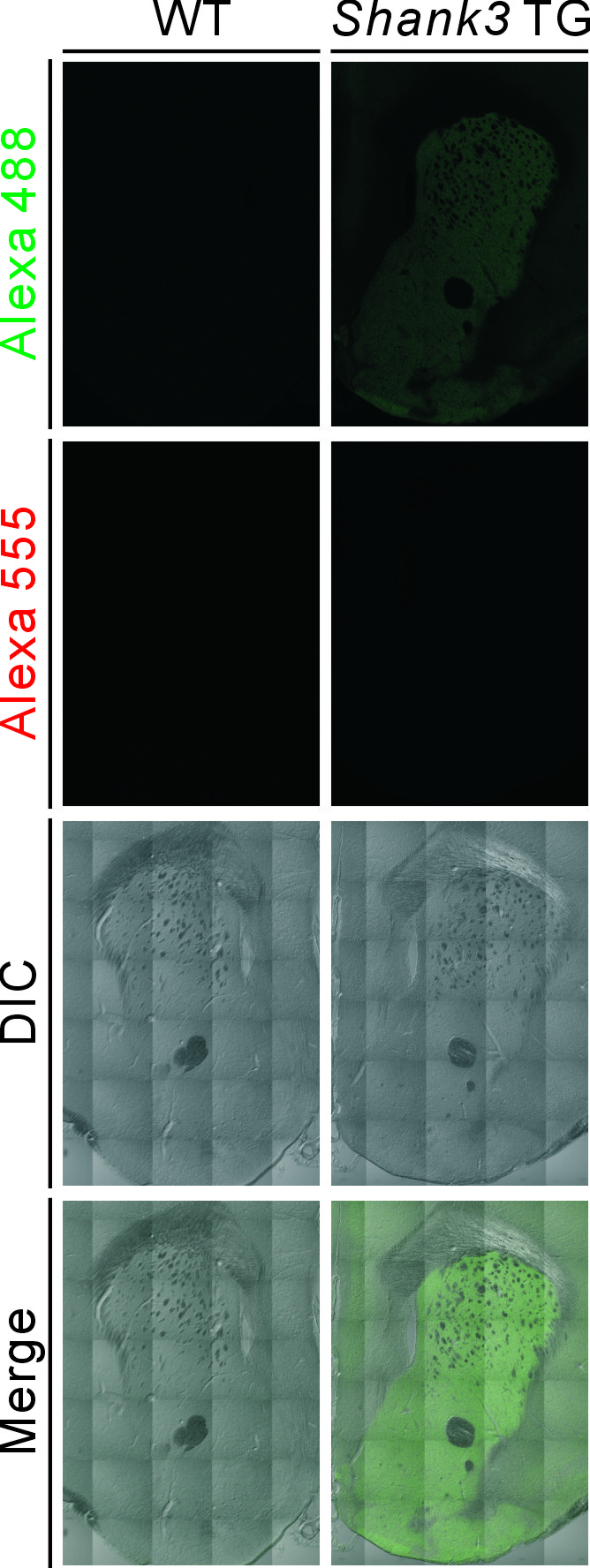

Supplement: FIGURE S2 — Detection of EGFP signal from the striatum of Shank3 TG mice. To test whether there was any background signal from the striatal sections of WT and Shank3 TG mice, we performed IHC experiments only with the secondary antibodies (Alexa Fluor 488 and 555). Under the scanning condition for Alexa Fluor 488, significant amount of signal (potentially from the EGFP-Shank3 proteins) was detected from the striatum of Shank3 TG, but not WT, mice. Meanwhile, no signal was detected for Alexa Fluor 555 from both WT and TG striatum. DIC, differential interference contrast. [file Image_2.jpeg]

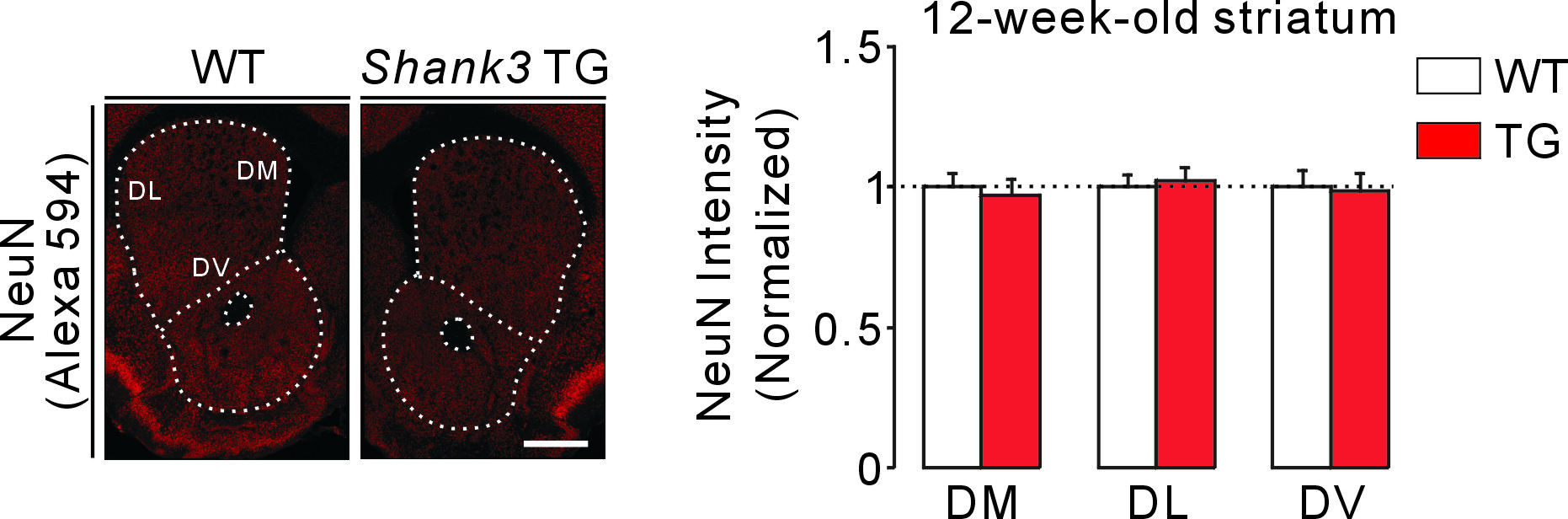

Supplement: FIGURE S3 — Normal NeuN intensity in the dorsal striatum of Shank3 TG mice. Representative IHC images and quantification show normal NeuN intensity in the DM, DL and DV compartments of Shank3 TG striatum. Scale bar, 500 μm. DL, dorsolateral; DM, dorsomedial; DV, dorsoventral. Data are presented as mean ± SEM (n = 5 animals per genotype; P > 0.05, unpaired two-tailed Student’s t-test). [file Image_3.jpeg]
